# Impact of the COVID-19 Pandemic Lockdown on Routine Childhood Immunization: A Saudi Nationwide Cross-Sectional Study

**DOI:** 10.3389/fped.2021.692877

**Published:** 2021-06-18

**Authors:** Leena R. Baghdadi, Afnan Younis, Hessah I. Al Suwaidan, Marwah M. Hassounah, Reem Al Khalifah

**Affiliations:** ^1^Department of Family and Community Medicine, College of Medicine, King Saud University, Riyadh, Saudi Arabia; ^2^Paediatric Endocrinology Division, Paediatrics Department, College of Medicine, King Saud University, Riyadh, Saudi Arabia

**Keywords:** COVID-19, pandemic, immunization, vaccination, children, Saudi Arabia

## Abstract

**Background:** Routine childhood immunization is the most cost-effective method to prevent infection and decrease childhood morbidity and mortality. The COVID-19 pandemic has affected access to health care in Saudi Arabia, including mandatory vaccinations for young children. We aimed to assess the prevalence of intentionally delayed vaccinations in children aged ≤ 2 years during the COVID-19 pandemic curfew in Saudi Arabia, its relation to the caregivers' fear of infection, and identifying factors affecting the caregivers' decision.

**Methods:** We conducted a cross-sectional study using a self-administered survey that targeted primary caregivers of children aged ≤ 2 years residing in Saudi Arabia during the COVID-19 pandemic curfew (March 4–July 6, 2020).

**Results:** We received responses from 577 caregivers, of whom 90.8% were mothers. The prevalence of intentional vaccination delay was 37%. Upon adjusting the potential confounders, the odds of delaying scheduled childhood vaccination because of COVID-19 pandemic fears were greater among caregivers with higher levels of fear (OR 1.10, 95% CI 1.02–1.11). Common reasons for delaying vaccinations were COVID-19 infection and prevention of exposure to COVID-19 cases.

**Conclusion:** Intentional vaccination delay leaves young children vulnerable to preventable infectious diseases. Identifying these children and offering catch-up vaccinations reduces this risk. Campaigns to increase awareness about the dangers of delaying vaccine-preventable diseases must be promoted to caregivers in addition to the promotion of home vaccination services. In preparation for future pandemics, we recommend countries consider interventions to control the level of fear and anxiety provoked by the pandemics and media, and interventions for improved access to vaccinations.

## Introduction

Immunization is considered the most cost-effective method to prevent infection and decrease childhood morbidity and mortality when administered as scheduled to sustain gained herd immunity (1). Delayed vaccination decreases vaccine coverage and can lead to outbreaks (2, 3). Pandemics have significant impacts on access to health care that leads to high rates of morbidity and mortality in a wide range of geographical areas (4). The spread of infectious diseases during a pandemic has been a cause of concern. The disrupted delivery of basic health services, including vaccination is a less-publicized consequence of pandemics.

In low- and middle-income countries, the COVID-19 pandemic is an important reason for delaying scheduled vaccinations (5, 6). A recent study conducted in a rural area in Africa highlighted the importance of vaccination during COVID-19 pandemic as showed decreased rate of children vaccinations (especially booster vaccines) (7). One week after the declaration of the national emergency, there was a significant decline in the number of vaccines ordered for children aged ≤ 2 years in the USA (5). The World Health Organization (WHO) reported at least 80 million children aged ≤ 1 year to be at risk of preventable diseases, including polio, diphtheria, and measles due to COVID-19-related disruptions of routine vaccinations (1).

In Saudi Arabia, childhood immunization is mandatory and neglect of childhood vaccination is considered a breach of the National Child Protection Law (8). Pediatric vaccination program provides primary vaccination series to prevent serious but preventable infectious diseases including tuberculosis, poliomyelitis, hepatitis A, hepatitis B, haemophilus influenzae type b, rota viral infection, measles, mumps, rubella, pertussis, diphtheria, tetanus, streptococcus pneumoniae, neisseria meningitidis, and varicella (9). Typically, parents get their children vaccinated free of charge at government clinics according to the vaccination schedule that follows the Ministry of Health (MOH) recommendations aligned with the World Health Organization (WHO) recommendations for childhood vaccinations (9). Intentional childhood vaccination delay was reported by 24% of parents before the pandemic (10). The common cited reasons for delayed vaccination are difficult access to health-care facilities, poor socioeconomic status, lower parental education, multiple parity (3, 11), and fear of vaccine safety and effectiveness (4).

During the pandemic, the Saudi health-care services rapidly transformed their organizations and workforce in response to the COVID-19 pandemic to minimize potential vaccination delays. For example, during the pandemic the use of a mobile application to promote vaccination and help immunization campaigns, and telehealth services to treat and monitor patients at home were initiated (12, 13). These telehealth services were combined with home visits for essential health services, including child vaccinations. The home vaccination services were provided by a few government tertiary hospitals and private hospitals (14). The access to home vaccination services was limited by the eligibility for those facilities, and lack of insurance coverage for vaccination consultations and prescriptions (15).

To date, there are few local observational studies about declines in vaccination rates during the pandemic. Two hospital-based studies conducted in Saudi Arabia reported decreased numbers of vaccination visits during the pandemic when compared with the previous 3 years (16, 17). Therefore, we aimed to estimate the nationwide prevalence of reported caregivers' intentional vaccination delay for children aged ≤ 2 years, assess its relation to the fear of the COVID-19 pandemic, the impact of home vaccination services, and potential predictors that can influence caregivers' vaccination decisions to enable future public health planning during pandemics.

## Materials and Methods

### Study Population and Design

We conducted a cross-sectional study using an electronic self-administered survey that targeted primary caregivers of children aged ≤ 2 years residing in Saudi Arabia, and excluded children with immune deficiency or individualized vaccination schedules. Participants were approached during the COVID-19 pandemic curfew (March 24–July 6, 2020) using the snowball method of sending invitations via social media (including Twitter, Snapchat, Facebook, mothers' WhatsApp groups) and local breastfeeding counseling groups, followed by a reminder message sent 2 days apart (18). This method was chosen because of the difficulty in accessing the target population during the lockdown, and because primary health-care assessment will be biased toward reporting the prevalence of vaccination delay among those who have access to those services during lockdown. The entire Saudi population had internet access free of charge during the lockdown. More than 89% of the Saudi population used the internet at that time (19).

#### Study Measures

The survey was developed in Arabic and English by experts: a pediatrician, who is also a methodologist and public health experts. The questions assessed the child and caregiver's demographics, socioeconomic status, health and health service-related factors, family unit, housing situation, administered vaccines, vaccination delay, intentions for vaccination delay, reasons for vaccination delay, accessibility for home vaccination, fear of COVID-19 infection, social media, pandemic news, peer pressure from family, friends and co-workers, social distancing, and home quarantine.

The fear of COVID-19 scale was translated into Arabic and validated (20). The parents' or caregivers' fears were estimated based on the fear paradigms, known as “fear-level classifiers.” Participants responded to phrases that measured their fear of the COVID-19 pandemic on a 5-point Likert-type scale. The answers included, “strongly disagree,” “disagree,” “neither agree nor disagree,” “agree,” and “strongly agree”; the possible scores were minimum 1 and maximum 5. A total score was calculated by adding the scores for each item. Higher scores indicated a severe fear level (20).

### Outcomes

The primary outcome was the prevalence of the caregivers' intentional delay of scheduled vaccines for children aged ≤ 2 years. Vaccination delay was defined as lack of vaccine administration at the required age of administration similar to the definition of vaccination delay measured in other studies conducted in Saudi Arabia (6, 10, 16, 17). The scale of delayed vaccine administration was dichotomized into “agree” and “disagree” statements. The vaccines reported by the caregivers were doubled checked and compared to the age of the child to make sure that we are including the correct missed vaccines per age group. The secondary outcomes included assessing the relationship between vaccination delay and fear of the COVID-19 pandemic, and assessing the potential predictors that could influence the caregivers' decisions about vaccination.

#### Ethical Considerations

We obtained approval for this study from the Institutional Review Board of King Saud University College of Medicine (Ethics Approval Number: E-20-4795). The study procedures complied with Good Clinical Practice and the Declaration of Helsinki. We obtained online consent from parents for ease of use and to maintain the confidentiality of the data.

### Power Sample Size and Statistical Analyses

Based on the population size of 2,674,932 children aged <4 years old residing in Saudi Arabia, of whom 1.7% are expatriate children (21), the prevalence of intentional childhood vaccination delay in Saudi Arabia was 24% (10); and assuming a response rate of 50 and 85% power (type-I error rate = 0.05), the sample size needed for the survey was 304 caregivers. We performed data analysis with the Statistical Package for Social Sciences v.27 (SPSS v.27, IBM Corp., New York, USA). We reported continuous data using means ± standard deviations (SD) for normally distributed data, medians and interquartile ranges for skewed data, and described categorical variables using frequencies and percentages. We calculated the social desirability bias as actual delayed vaccination prevalence—the parent-reported delayed vaccination prevalence. Using multivariate logistic regression we reported the prevalence of intentional vaccination delay adjusted for fear of the COVID-19 pandemic and after adjusting for potential confounders, including sociodemographic factors, caregivers' and children's ages and chronic diseases, health-care factors, scheduled vaccination history of older siblings, and COVID-19 factors [exposure to COVID-19, admission to the intensive care unit (ICU), and fear of COVID-19). Further analysis was conducted to explore whether the parents and/or caregivers sought more information to aid their vaccination-delay decision.

## Results

Of the 577 respondent caregivers, 90.8% were mothers, and 93.6% were Saudi citizens (Table 1). The caregivers' mean age was 32.6 ± 5.7 years, and mean parity was 2.6 ± 1.5 children per family. The majority of caregivers had no chronic illness, high socioeconomic status (earning Saudi Arabian riyal 10,000/month), and a high educational level. The majority of caregivers lived in Riyadh (61.5%), followed by the Makkah (15.3%) and Madinah (6.8%) regions. The mean age of the youngest child was 16.5 ± 11.1 months and the mean age of the sibling was 17.5 ± 10 months. The majority of children (85.79%) had no chronic illness, 48.2% of the respondents had private health insurance, and 48.5% of them received their vaccinations at primary health-care centers. Participants who knew someone diagnosed with COVID-19 were 39.9% and 1.4% of participants themselves had COVID-19. On average, caregivers spent 11.2 ± 10.6 h/week on social-media platforms.

**Table 1:** Characteristics of the parents or primary caregivers, children, and their health care.

	***N* (%)**	**Mean (SD)**
**PARENTS OR PRIMARY CAREGIVERS' CHARACTERISTICS**		
**Age of caregiver (years)**	577	32.6 (5.7)
**Caregivers**		
Mothers	524 (90.8)	
Fathers	40 (6.9)	
Grandparents	13 (2.3)	
Number of family members		4.5 (1.7)
Number of children		2.6 (1.5)
Nationality, Saudi	540 (93.6)	
**Residential region of the caregivers and children**		
Riyadh	355 (61.5)	
Easter	48 (8.3)	
Najran	1 (0.2)	
Qassim	3 (0.5)	
Hail	7 (1.2)	
Makkah	88 (15.3)	
Baha	1 (0.2)	
Asir	10 (1.7)	
Jazan	5 (0.9)	
Madinah	39 (6.8)	
Tabuk	4 (0.7)	
Others	16 (2.8)	
**Caregiver's education level**		
Elementary school	2 (0.3)	
Secondary school	4 (0.7)	
High school	45 (7.8)	
Diploma	31 (5.4)	
Undergraduate degree	341 (59.1)	
Postgraduate degree or PhD	154 (26.7)	
**Marital status**		
Married	559 (96.9)	
Widowed	4 (0.7)	
Divorced or separated	11 (1.9)	
Single	3 (0.5)	
**Monthly income [Saudi Arabian riyal (SAR)]**		
≥SAR 10,000	368 (63.8)	
SAR 5,000 to SAR 9,000	169 (29.3)	
< SAR 5,000	40 (6.9)	
Caregiver's working status, working	330 (57.2)	
**Caregiver's chronic diseases**		
No chronic illness	431 (74.7)	
Obesity	33 (5.7)	
Diabetes	18 (3.1)	
Asthma	49 (8.5)	
Hypertension	20 (3.5)	
Autoimmune disease	5 (0.87)	
Multiple sclerosis	6 (1.03)	
High cholesterol	15 (2.6)	
COVID-19 fear score	^*^21.98 (5.5)	
Caregivers with a previous diagnosis of COVID-19	8 (1.4)	
Knowing someone admitted to the intensive care unit due to COVID-19	30 (2.2)	
Knowing someone with confirmed COVID-19	230 (39.9)	
**Relation to a confirmed COVID-19 case**		
Friend	101 (48.8)	
Own colleague or spouse's colleague	60 (29.0)	
Close family	34 (16.4)	
Domestic help	6 (2.9)	
Immediate family	6 (2.9)	
Average hours/week spent on social-media platforms	11.2 (10.6)	
**DEMOGRAPHIC CHARACTERISTICS OF THE CHILDREN**		
Age of the youngest child (in months)		^#^14 (8)
Age of the oldest child (in months)		^#^19 (8)
**Chronic diseases in the children**		
No chronic diseases	495 (85.79)	
Born prematurely	33 (5.72)	
Diabetes	12 (2.1)	
Syndromes	2 (0.34)	
Metabolic diseases	4 (0.70)	
Heart diseases	6 (1.04)	
Asthma	9 (1.56)	
Lung diseases	3 (0.52)	
Genetic diseases	8 (1.38)	
Hypothyroidism	2 (0.34)	
Growth issues	3 (0.51)	
Private health insurance	278 (48.2)	
**Place of vaccination**		
Primary-care center or clinic	280 (48.5)	
Government hospital	118 (20.5)	
Private hospital	179 (31.0)	

*SD, standard deviation;*

* *minimum fear score was 7 and maximum was 35;*

# *Median and interquartile range*.

When the standard childhood vaccination schedule for Saudi Arabia is considered 37% of parents had delayed vaccinating their children during the pandemic, and only 21% of these parents acknowledged delayed vaccination (Table 2). When evaluating the difference between the reported population vaccination delay previously and our findings the OR is 1.77 *P*-value < 0.0001. The social desirability bias was 16 and 19% of parents had delayed vaccination for their elder child. Vaccination delays were highest for 9-month (19% of children) and 24-month (22% of children) vaccinations. The fear level of COVID-19 among parents increased the prevalence of delayed vaccination (OR 1.03, 95% CI 0.99-1.06, *p*-value = 0.05). This relationship increased after adjusting for confounders [caregivers' and children's ages and chronic diseases, scheduled vaccination history for older siblings, and COVID-19 factors (exposure to COVID-19, admission to ICU, and fear of COVID-19)] (OR 1.10, 95% CI 1.02–1.11, *p*-value = 0.006).

**Table 2:** Children's vaccination status.

	***N* (%)**
**Younger child**	577
**Scheduled vaccinations reported by caregivers**	
Up-to-date	451 (78.2)
Not up-to-date	126 (21.8)
**Received vaccinations based on the child's age**	
Up-to-date	366 (63)
Not up-to-date	211 (37)
**Missed (incomplete)** ^*^vaccinations at	
Birth	3 (1)
2 months	15 (5)
4 months	36 (12)
6 months	40 (14)
9 months	55 (19)
12 months	46 (16)
18 months	35 (12)
24 months	65 (22)
**Older child**	**67**
**Scheduled vaccinations reported by caregivers**	
Up-to-date	**57 (85)**
Not up–to-date	**10 (15)**
**Received vaccinations based on the child's age**	
Up-to-date	54 (80.5)
Not up-to-date	13 (19.4)
**Missed (incomplete)** ^*^vaccinations for older child at	
Birth	0 (0)
2 months	3 (23)
4 months	2 (15)
6 months	3 (23)
9 months	3 (23)
12 months	0 (0)
18 months	0 (0)
24 months	2 (15)

* *Vaccination schedule in Saudi Arabia at Birth: 1^st^ dose of hepatitis B vaccine (HepB); 2 months: 2^nd^ dose of HepB, 1^st^ dose of rotavirus vaccine (Rota), 1^st^ dose of diphtheria, tetanus, and acellular pertussis combination vaccine (DTaP), 1^st^ dose of Haemophilus influenzae type b vaccine (Hib), 1^st^ dose of pneumococcal conjugate vaccine (PCV), and 1^st^ dose of inactivated poliovirus vaccine (IPV); 4 months: 3^rd^ dose of HepB, 2^nd^ dose of Rota, 2^nd^ dose of DTaP, 2^nd^ dose of Hib, 2^nd^ dose of PCV, and 2^nd^ dose of IPV; 6 months: 4^th^ dose of HepB, 3^rd^ dose of DTaP, 3^rd^ dose of Hib, 3^rd^ dose of PCV, 2^nd^ dose of IPV, 1^st^ dose of oral poliovirus vaccine (OPV), and BCG vaccine; 9 months: 1^st^ dose of quadruple conjugated meningococcus vaccine (MCV4) and 1^st^ dose of measles vaccine; 12 months: 1^st^ dose of measles, mumps, rubella vaccine (MMR), 2^nd^ dose of MCV4, 2^nd^ dose of OPV, and 4^th^ dose of PCV; 18 months: 4^th^ dose of DTaP, 4^th^ dose of Hib, 2^nd^ dose of OPV, 1^st^ dose of HepA, 1^st^ dose of Varicella, 2^nd^ dose of MMR; 24 months: 2^nd^ dose of HepA*.

*The bold value (67) is the total (N) number of older children*.

*The bold value [57 (85)] is the N (%) of older children who were Up-to-date with vaccinations*.

*The bold value [10 (15)] is the N (%) of older children who were Not Not up-to-date with their vaccination*.

The mean fear of COVID-19 was 21.98 ± 5.5 points (minimum 7 and maximum 35 points). The intention and vaccination decision were significantly impacted by the mean fear level (Table 3). About 41% of caregivers never thought of delaying vaccinations, 30% considered delaying vaccinations, yet followed the vaccination schedule, 2.4% decided to delay vaccinations; however, they could not delay them for any specific reason; and 26.9% did delay vaccinations.

**Table 3:** Fear level of COVID-19 stratified by the decision about vaccination (*N* = 577).

**Responses**	***N* (%)**	**Mean**	**SD**	***P*-value**
Never thought of delaying vaccination	235 (40.7)	22.21	5.674	<0.001^*^
I thought about delaying the vaccination, but still vaccinated at the scheduled time	173 (30)	22.33	4.869	
I decided to delay the vaccination, but I could not find a reason to do so	14 (2.4)	18.43	4.052	
I delayed the vaccination	155 (26.9)	21.55	5.899	

*SD, standard deviation;*

* *p-value is significant at 0.05*.

After adjusting for caregivers' and children's ages, chronic diseases, history of receiving scheduled vaccination for the older siblings, and COVID-19 factors: exposure to COVID-19, admission to the intensive care unit, and fear level of COVID-19, other factors that lead to vaccination delay are concerns about the safety and efficacy of vaccines, the caregivers were afraid they or their children would contract COVID-19, concerns of vaccine safety and effectiveness, the family was traveling within Saudi Arabia, no available transportation or access to a health-care facility, curfew timings were tight (Table 4). None of the caregivers listed the overload of work with COVID-19-cases in the hospitals and the offices as a reason for denying the vaccination appointment.

**Table 4:** Unadjusted and adjusted regression models for reasons for delayed vaccination.

**Reasons**	**Unadjusted model**			^**^Adjusted model		
	**^**#**^OR**	***P*-value**	**95% CI**	**^**#**^OR**	***P*-value**	**95% CI**
Afraid that my child will get infected with COVID-19	3.56	>0.001^*^	2.44–5.19	4.14	>0.001^*^	2.74–6.27
Afraid of getting infected with COVID-19	2.86	>0.001^*^	1.97–4.16	3.34	>0.001^*^	2.19–5.09
Concerns of vaccine safety and effectiveness	4.19	>0.001^*^	2.09–8.41	4.68	>0.001^*^	2.14–10.27
I was traveling within Saudi Arabia	4.99	0.009^*^	1.48–16.8	4.52	0.02^*^	1.26–16.22
No transportation or access to a health-care facility	3.31	0.029^*^	1.13–9.7	3.04	0.059	0.96–9.61
Curfew timings were tight	3.43	>0.001^*^	2.15–5.47	4.57	>0.001^*^	2.73–7.66
Other	1.15	0.61	0.68–1.95	1.23	0.48	0.70–2.16

*CI, confidence interval; OR, odds ratio;*

# *Reference group= did not delay the vaccine;*

* *p-value is significant at 0.05;*

** *Model adjusted for caregivers' and children's ages, chronic diseases, history of receiving scheduled vaccination for the older siblings, and COVID-19 factors: exposure to COVID-19, admission to the intensive care unit, and fear level of COVID-19*.

Eighty two percent of parents knew about the home vaccination service and 17.5% of them used the service during the lockdown. The reasons for not using the home visit vaccination service, included not knowing about it (17.9%), not needing the service to-date (16.3%), their hospital facility not providing it (13.8%), preference for clinic visits (12%), expense (7.4%), doubts about home visit vaccinations (7.1%), and fear of COVID-19 infection (7.1%) (Supplementary Table 1).

When assessing the impact of these reasons on delayed vaccination, after adjusting for caregivers' and children's ages, chronic diseases, history of receiving scheduled vaccination for older siblings, exposure to COVID-19, admission to the intensive care unit, and fear level of COVID-19, only two reasons were statistically significant. These were not knowing about the home care vaccination service and fear of contracting COVID-19 during vaccination (OR = 2.51 and OR = 3.45, respectively) (Table 5). Those parents who preferred clinic visits for vaccination were the least likely to delay vaccination (OR = 0.23, *p*-value = 0.002).

**Table 5:** Reasons for not using the home-visit vaccinations and delaying vaccination: unadjusted and adjusted regression models.

**Reasons**	**Unadjusted model**			^**^Adjusted model		
	**^**#**^OR**	***P*-value**	**95% CI**	**^#^OR**	***P*-value**	**95%CI**
I do not know how to call or reach the clinic	1.97	0.15	0.79–4.97	1.95	0.19	0.71–5.35
Clinic staff were not very cooperative when scheduling appointments	0.94	0.92	0.28–3.16	1.30	0.69	0.35–4.82
No home visit was provided by the government hospital	0.80	0.49	0.43–1.50	0.71	0.34	0.35–1.4
I did not have any idea about home-visit vaccinations	2.01	0.008	1.20–3.37	2.51	0.003^*^	1.37–4.58
Not needed until now	0.48	0.031^*^	0.25–0.93	2.58	0.014^*^	1.21–5.47
I am afraid of getting infected	3.06	0.005^*^	1.41–6.65	3.45	0.005^*^	1.45–8.18
I do not trust the quality of the home-visit vaccinations service	1.65	0.21	0.76–3.58	1.65	0.25	0.70–3.88
I prefer vaccinations at my child's doctor's clinic	0.23	0.002^*^	0.09–0.59	0.18	0.002^*^	0.06–0.54
Expensive service	1.802	0.13	0.84–3.85	2.13	0.08	0.91–5.03
It is not covered by my health insurance	0.48	0.26	0.14–1.72	0.45	0.24	0.12–1.68

*CI, confidence interval; OR, odds ratio;*

# *Reference group = did not delay the vaccine,*

* *p-value is significant at 0.05;*

** *Model adjusted for caregivers' and children's ages, chronic diseases, history of receiving scheduled vaccination for older sibling, and COVID-19 factors: exposure to COVID-19, and admission to the intensive care unit; the model is also adjusted for fear level of COVID-19 expect for one reason “I am afraid of getting infection” to avoid adjusting for COVID-19 fear twice*.

## Discussion

Interrupted routine childhood vaccination can lead to outbreaks of preventable infections. The COVID-19 pandemic negatively impacted children's scheduled vaccinations in Saudi Arabia. The prevalence of intentional vaccination delay increased from 24% in earlier reports (6) to 37% during the COVID-19 lockdown. Delayed vaccination was more noticeable among the caregivers with higher COVID-19 fear levels, and for the 9-month measles and meningococcal conjugate quadrivalent vaccine, and the 24-month hepatitis A vaccine.

Although the majority of our respondents were of higher socioeconomic status and had high education levels that is similar to the general population, the fear of potentially exposing themselves or their children to COVID-19 during well-child visits and vaccination safety during the pandemic had a major negative impact on the caregivers' decision to vaccinate children. The increased rate of vaccination delay among our respondents is alarming with even higher rates of delayed vaccinations among the lower socioeconomic status caregivers and those with no social media access. This vaccine hesitancy could be the influence of public misinformation, and belief in vaccine safety and efficacy during the pandemic (22, 23). Our nationwide findings are similar to the two Saudi hospital-based studies reporting delayed child vaccinations during COVID-19 (24, 25). This is similar to the decreased rate of vaccination in the USA (where parents avoided visiting clinics or hospitals to avoid exposure to COVID-19) and other developed countries (5, 24, 26). Many parents canceled their children's appointments leading to a 75% drop in vaccination rates (25). Historically, vaccination delays have been reported during previous pandemics [2009–2010 swine flu (H1N1) pandemic and 2015–2016 Zika virus outbreak]; increased fear of the pandemic and concern for loved ones' health were the most-reported concerns (24).

Deaths prevented by supporting routine childhood immunizations outweigh the excess risk of deaths from COVID-19 due to visiting vaccination clinics. For every 1 excess COVID-19 death attributable to the COVID-19 infections acquired during routine vaccination clinic visits, 84 deaths (95% CI 14-267) in children could be prevented by sustaining routine childhood immunization (27), especially in in rural areas (7). This reinforces the importance of the WHO guidelines emphasizing routine childhood immunization schedules to continue despite outbreaks and pandemics (28, 29). If the fear of COVID-19 or any future pandemics triggered a breakdown of the vaccination systems, child mortality caused by vaccine-preventable diseases could increase significantly (23). Therefore, public health efforts as well as the media should focus on reinforcing benefit-risk ratios for routine childhood immunizations and access to obtain health maintenance rather than acute care.

While home vaccination services were not widely available in Saudi Arabia, the lack of availability and knowledge about the service had predicted childhood vaccination delay (30). Policy makers, pediatricians, and primary care physicians need to consider offering these services, and promoting the utilization of available services in the community, in addition to implementing public health strategies to reduce the number of parents at the pre-contemplation and contemplation stage of vaccination delay. In our survey, only 40% of parents did not think about delaying vaccination while 32% were in the early stages of behavioral change. The findings of this study can inform future national emergency and crisis-preparedness planning for basic ongoing services.

The included sample is comparable to the Saudi general population, and the results were adjusted for minority groups such as children from low income, and lower educational level families. Although we received an adequate sample size, we had expected a higher response rate. The low response rate reflected the non-response bias associated with online surveys. This was expected especially during the COVID-19 pandemic as many people experienced higher psychological stress, in addition to the increased number of online surveys leading to survey fatigue. We used the non-probability sampling technique because the whole nation was under community-containment measures and asked to remain home, except for necessary trips. We opted for snowball sampling through social media.

During the early phase of the pandemic lockdown and the implementation of strict social distancing measures, it was difficult to access children's vaccination records since vaccination records are not documented on a national electronic health data base nor caregivers were able to attach a copy of document to have an objective measure of vaccination delay. Therefore, we used the parental report of vaccination delay, which is an acceptable way of measuring outcomes in pediatrics (31) although it is susceptible to biases such as recall bias, and social desirability bias (32). Responses to the vaccination-related questions are prone to a social desirability bias (32). In our survey, 16% of parents had delayed their children's vaccinations from the Saudi vaccination schedule but said that vaccination was up-to-date for their child. However, we tried to minimize the response bias by online self-administration, avoiding questions on responder identity, and contact information. Future studies are needed to determine the prevalence of vaccine delay based on verification of the child's vaccination records, sampling by the child's zip code, and over sampling for children from low socioeconomic status families, expatriates, and assess the impact of the reported decline in vaccination on the population's herd immunity.

The COVID-19 pandemic negatively impacted young Saudi children's scheduled vaccinations because of the fear of COVID-19 infection. Identifying these children and offering them the missed vaccinations can decrease their risk of common childhood diseases. Campaigns to increase awareness about the dangers of delaying vaccine-preventable diseases must be promoted to caregivers, as well as the promotion of home vaccinations services. In preparation for future pandemics, we recommend that countries consider interventions to control the level of fear and anxiety provoked by the pandemics and media, and interventions for improved access to vaccinations.

## Data Availability Statement

The original contributions presented in the study are included in the article/Supplementary Material, further inquiries can be directed to the corresponding author/s.

## Ethics Statement

The studies involving human participants were reviewed and approved by Institutional Review Board of King Saud University College of Medicine (Ethics Approval Number: E-20-4795). The patients/participants provided their written informed consent to participate in this study.

## Author Contributions

LB formulated the research questions, created the study design, carried out the study, managed and analyzed the data, assisted in writing the results, created the first draft of the manuscript, and finalized the manuscript. AY helped formulate the research question, assisted in the study design, writing of the results, and read and approved the manuscript. RA helped formulate the research question, assisted with the study design, conducted the pilot testing, and read and approved the manuscript. HA performed data cleaning, assisted with data coding, conducted data analyses, and read and approved the manuscript. MH assisted with formulating the research questions, study design, and read and approved the manuscript. All authors contributed to the article and approved the submitted version.

## Conflict of Interest

The authors declare that the research was conducted in the absence of any commercial or financial relationships that could be construed as a potential conflict of interest.
