# Supplementary material for: Impact of the COVID-19 Pandemic Lockdown on Routine Childhood Immunization: A Saudi Nationwide Cross-Sectional Study
Source: Front Pediatr. 2021 Jun 18;9:692877. doi: 10.3389/fped.2021.692877 (PMC8249725; doi:10.3389/fped.2021.692877)
Supplement: Supplementary file 1 [file Table_1.DOCX]

**Table S1. Using the home-visit vaccinations and the reasons for not using it.**

|  | N (%) 577 |
| --- | --- |
| **Used home visit vaccinations** |  |
| Yes | 101 (17.5) |
| No | 476 (82) |
| **Reasons for not using home visits** |  |
| I was not aware about home visit vaccinations | 85 (17.9) |
| Not needed until now | 78 (16.3) |
| No home visit provided by government hospital | 65 (13.8) |
| I prefer taking vaccines with my child doctor at the clinic | 57 (12) |
| Expensive service | 35 (7.4) |
| I do not trust the quality of home visit vaccinations service | 33 (7.1) |
| I am afraid of getting infected | 34 (7.1) |
| My child next vaccine appointment is not yet | 26 (5.4) |
| I do not know how to call or reach | 24 (5) |
| It is not covered by health insurance | 18 (3.7) |
| They were not very cooperative when scheduling appointments | 14 (2.9) |
| No pandemic during those times | 7 (1.4) |
